# A Thermolabile Phospholipase B from *Talaromyces marneffei* GD-0079: Biochemical Characterization and Structure Dynamics Study

**DOI:** 10.3390/biom10020231

**Published:** 2020-02-04

**Authors:** Rabia Durrani, Faez Iqbal Khan, Shahid Ali, Yonghua Wang, Bo Yang

**Affiliations:** 1 School of Biology and Biological Engineering South China University of Technology 382 East Outer Loop Rd, University Park, Guangzhou 510006, China; rabiadurrani_kust@yahoo.co.uk; 2 School of Electronic Science and Engineering, University of Electronic Science and Technology of China, Chengdu 610054, China; khanfaeziqbal@gmail.com; 3 School of Food Science and Engineering, Guangdong Research Center of Lipid Science and Applied Engineering Technology, State Key Laboratory of Pulp and Paper Engineering, South China University of Technology, Guangzhou 510641, China; ali.ali.md111@gmail.com (S.A.); yonghw@scut.edu.cn (Y.W.)

**Keywords:** free fatty acids (FFAs), NMR (nuclear magnetic resonance), phospholipase B, *Talaromyces marneffei*, affinity chromatography

## Abstract

Phospholipase B (EC 3.1.1.5) are a distinctive group of enzymes that catalyzes the hydrolysis of fatty acids esterified at the *sn-1* and *sn-2* positions forming free fatty acids and lysophospholipids. The structural information and catalytic mechanism of phospholipase B are still not clear. Herein, we reported a putative phospholipase B (TmPLB1) from *Talaromyces marneffei* GD-0079 synthesized by genome mining library. The gene (TmPlb1) was expressed and the TmPLB1 was purified using *E. coli* shuffle T7 expression system. The putative TmPLB1 was purified by affinity chromatography with a yield of 13.5%. The TmPLB1 showed optimum activity at 35 °C and pH 7.0. The TmPLB1 showed enzymatic activity using Lecithin (soybean > 98% pure), and the hydrolysis of TmPLB1 by ^31^P NMR showed phosphatidylcholine (PC) as a major phospholipid along with lyso-phospholipids (1-LPC and 2-LPC) and some minor phospholipids. The molecular modeling studies indicate that its active site pocket contains Ser125, Asp183 and His215 as the catalytic triad. The structure dynamics and simulations results explained the conformational changes associated with different environmental conditions. This is the first report on biochemical characterization and structure dynamics of TmPLB1 enzyme. The present study could be helpful to utilize TmPLB1 in food industry for the determination of food components containing phosphorus. Additionally, such enzyme could also be useful in Industry for the modifications of phospholipids.

## 1. Introduction

Phospholipase B (EC 3.1.1.5) are the unique group of enzymes that catalyzes the hydrolytic cleavage of fatty acids that are esterified both at the *sn-1* and *sn-2* positions. The hydrolysis reaction produces free fatty acids (FFAs) and lysophospholipids or glycerol-3-phosphodiesters [1,2,3]. Phospholipase B isolated from several fungi such as *S. cerevisiae*, *C. albicans, C. utilus*, *P. chrysogenum,* and *C. neoformans* showed hydrolase and acyltransferase activity. They showed high preference for substrate from lysophospholipids to diacylphospholipids and hydrolyzes diacylphospholipids without forming lysophospholipids. Some of the phospholipase B enzyme having lysophospholipase–transacylase activity which transfers free fatty acids to lysophospholipids and forms diacylphospholipid [4]. These enzymes are mostly found in animals and microbes. In animals, they are present in mammalian tissues and venom and have a potential role as virulence. They are also found in amoeba, bacteria, pathogenic, and nonpathogenic fungi. Their enzymatic activity has also been detected in some plants [1,5].

The structural features and catalytic mechanism showed that these enzyme contain C-terminal hydrophobic sequence with an extracellular domain and a signal peptide for secretion. They are usually glycosylated and termed as ecto-phospholipases. In animals, they possess huge extracellular domains that has four trails of similar repeats (I–IV) having II–IV with various GDSL and GXSXG motifs. The *serine* residues in domain II has the phospholipase B activity [1,6]. The GXSXG consensus sequence discovered in fungal phospholipase B have *Arg*, *Ser,* and *Asp* as catalytic triad [1,7].

The phospholipase B from different species prefers phosphatidylcholine as substrate, but PC also have an important action on lyso-phospholipids without any particular priority [1,8]. This substrate preference is important for industrial application, in food industry and oil degumming employing phospholipase B. Furthermore, the high selection to use phosphotiylcholine as substrate is the production of artificial phospholipids or the phospholipids that are not common in nature. Since phosphotiylcholine is abundantly found in nature and is used a starting material for industrial and research applications [1,9].

The large variety of Phospholipase B from fungi such as *Saccharomyces cerevisae*, *Fusarium oxysporum*, *Aspergillus fumigatus*, *Cryptococcus neoformans,* and *Cryptococcus gattii* have been reported to be expressed, characterized, and their applications mainly as oil degumming have been studied [1,10,11,12]. Apart from oil degumming as an important application, phospholipase B are also used to change or transform phospholipids. The change or modification includes hydrolysis of phospholipids, synthesis of particular phospholipids and changing one form of phospholipid to another. Such kind of hydrolysis reaction involves free fatty acids (FFAs) of phospholipids using phospholipase B [1].

Low temperature phospholipase B enzyme could be more economic and environmentally friendly. In the food industry they are preferred as they avoid changes in food ingredients. Although, vast applications of low temperature phospholipase B could be studied but this area has not been touched by most of the researchers as compared to thermotolerant phospholipases. These isolates shows optimal activity from 4–37 °C and gets inactive at 40–50 °C [1].

In the present work, a phospholipase B from *Talaromyces marneffei* TmPLB1 was synthesized and cloned. Further, the biochemical characterization and classification of various phospholipids was carried out using ^31^P NMR to describe the potential use of this enzyme in food industry for the analysis of phosphorus compounds in foods at low temperature. We also performed structural modeling and dynamics studies to check the catalytic triads and conformational profile of TmPLB1. The overall secondary structure of TmPLB1 was found to be flexible and stable at its optimum conditions. This study establishes a primary foundation for understanding the role of low temperature phospholipase B for Industrial application.

## 2. Material and methods

### 2.1. Reagents and Chemicals

*Escherichia coli* (*E. coli*) Top10 and plasmid pET-28a (+) (Invitrogen, Shanghai, China) were used as cloning host and vector in this study. *E. coli* Shuffle T7 express competent cells (Invitrogen, Shanghai China). A SanPrep Column plasmid mini-preps kits were purchased from Sangon Biotech (Shanghai, China) to extract plasmid. Bradford protein assay kit (Sangon Shanghai, China) was used for measurement of protein concentration. The Molecular protein marker (code No: 3595Q) was purchased from TaKaRa Biotechnology Co. Ltd. Dalian, China. Isopropyl β-d-1-thiogalactopyranoside (IPTG) (TaKaRa Biotechnology Co. Ltd. Dalian, China). Lecithin (Soybean PC > 98%), CDCl_3_, Methanol (Aladdin Industrial corporation #1008 Qigang Rd, Nanqiao Town, Fengxian Shanghai (201406), China). Triphenyl Phosphate (TPP, purity 99.9%) (Shanghai Macklin Biochemical Co. Ltd., Shanghai, China). Other chemicals and reagents used in present study were of analytical grade.

### 2.2. Cloning, Expression and Purification of TmPLB1

The putative TmPlb1 gene sequence ATCC 18,224 (Accession XP_002148877) was taken from the genomic data of *Talaromyces marneffei* GD-0079 a dimorphic fungi. The gene was artificially synthesized by Sangon Biotech (Shanghai, China). Furthermore, TmPlb1 gene was cloned into pET-28a (+) vector (Gene Copoeia Inc, Rockville, MD, USA) in the location between *EcoRI* and *XhoI* and fused with C terminal His tag. The plasmid pET-28a (+) TmPlb1 (Sangon Biotech, Inc., Shanghai, China) was used as template and the plasmid containing the gene TmPlb1 was further transformed into *E. coli* Shuffle T7 for protein expression. *E. coli* Shuffle T7 cells were grown at 37 °C in *Luria-Bertani* broth (1% Tryptone, 0.5% NaCl, and 0.5% Yeast extract) by kanamycin 50mg/mL), until the optical density (OD_600_) reached up to 0.6–0.8. Isopropyl β-d-1-thiogalactopyranoside (IPTG) initiated protein induction with 0.1 mM concentration, till 22 h at 20 °C at 200 rpm with shaking. The cells were harvested by centrifugation at 12,000 rpm for 20 min and lysed by ultrasonication in phosphate buffer saline (PBS) (137 mM NaCl, 2.7 mM KCl, 10 mM Na_2_HPO_4_, 2 mM KH_2_PO_4_, pH 7.4). The sonication was done at 4 s with 4 s interval for 15 min. Cells were further centrifuged at 10,000× *g* for 30 min to eliminate impurities and the lysate was analyzed prior purification. The cell lysate was filtered with 0.45 µm filter and applied to a His prep^TM^ FF 16/10 (GE Healthcare, Uppsala, Sweden). The target protein was eluted with elution buffer B (500 mM Imidazole pH 7.4) and the purified protein was stored in Buffer A (20 mM Imidazole pH 7.4). The protein purification were analyzed by 12% SDS-PAGE (prepared in lab) and the protein concentration was analyzed by BCA protein assay kit from Sangon Biotech (Shanghai, China) according to the instructions provided in the manual of kit [13].

### 2.3. Biochemical Characterization of TmPLB1

#### 2.3.1. Hydrolytic Assay

The hydrolytic assay was performed in a 50 mL conical flask to check activity of protein at 200 rpm. The hydrolysis of TmPLB1 using Soy PC (Lecithin) emulsion (PC 4 g, Triton X-100 25 g (*w*/*v*) and H_2_O to make volume 100 mL using 20 mM PB, pH 7.0, purified enzyme (0.5 mg/mL) at (35 °C) for 10 min, with the addition of 15 mL 95% ethyl alcohol to stop the hydrolysis reaction. The fatty acids were detected by titration with 50 mM NaOH [14]. The controls were measured by heating the enzyme at 100 °C to make it inactive for 15 min. After cooling to a certain temperature, it was used as described for active enzyme. One unit is defined as the amount of enzyme that released one micromole of fatty acids in one minute [15].

#### 2.3.2. Effect of Temperature on TmPLB1 Activity

The optimum temperature for enzyme analysis were assessed using Soy PC (Lecithin) emulsion as substrate in 20 mM PB buffer at pH 7.0. The temperatures were selected from 20 to 55 °C [14]. The thermal stability of TmPLB1 were investigated by incubation of enzyme at different temperature including 30 °C, 35 °C, and 40 °C. At every 30 min, the samples were taken to check the relative activity.

#### 2.3.3. Effect of pH on TmPLB1 Activity

The pH for enzymatic activity were analyzed in different buffers 100 mM citric acid-sodium citrate (pH 4.0 and 5.0), 100 mM phosphate buffer (pH 6.0, 7.0), 100 mM Tris-HCl (pH 8.0), and 100 mM Gly-NaOH (pH 9.0) with pH from 4.0 to 9.0 and reacted at 35 °C. The stability of pH were investigated by pre-incubating the purified TmPLB1 in buffers with pH values range of 5–9 for 12 h at 4 °C. Aliquots were then withdrawn, and relative enzymatic activity was determined [14].

#### 2.3.4. Effect of Chemicals on TmPLB1 Activity and Stability

Metal ions (CsCl, KCl, MnCl_2_, FeCl_3_, NiCl_2_, NaCl, CaCl_2_, ZnCl_2_, Al_2_(SO_4_)_3_, and CuSO_4_) were used at 1 mM concentration using pH 7.0, and the enzyme stability were investigated by incubation at 4 °C for 2 h [15]. Detergents (1% and 5% *w*/*v*) such as Triton X-100, Tween-20, Tween-80, and SDS and organic solvents with a concentration of 50% (*v*/*v*) in the enzyme solution containing methanol, ethanol, 1-propanol, 2-propanol, and acetone were employed to check enzyme stability after coincubation for 2 h at 4 °C using pH 7.0. The measurement without adding chemicals was taken as 100% (control).

#### 2.3.5. Circular Dichroism Spectroscopy Analysis

Circular dichroism measurements were performed by a Chirascan^TM^ Ultra-sensitive spectroscopy Jasco *J* -815 (Applied Photo physics, Surrey, UK) with a Peltier temperature controller and single cuvette holder. Conformational changes in the secondary structure of protein were monitored in the far-UV region between 190 to 260 nm with a protein concentration of 0.5 mg/mL (300 μL) in a quartz cuvette with a path length of 1 mm. The solution conditions were 20 mM sodium phosphate buffer, pH 7.4. All measurements were carried out at 25 ± 0.1 °C. Three accumulations of successive scans were taken (within 600 HT voltage range) and averaged to get the complete spectra. The raw data at a given wavelength, λ (nm) were converted into concentration-independent parameter [θ]_λ_ (deg cm is the mean residue weight of the protein, *l* is the path length of the cell in centimeters, and *c* is the protein concentration in mg ml^−1^). Baseline was corrected and smoothed (within permissible limits) by using the inbuilt CDNN software (Applied Photo physics, Surrey, UK) of the unit [16,17,18].

### 2.4. Analysis of Phospholipids in Lecithin Using ^31^P NMR

^31^P NMR comprising solid and liquid state are selective analytical procedure for the detection and quantification of phosphorus in samples. It uses different chemical settings to differentiate various types of phospholipids. The samples were prepared by adding 3 g of Lecithin (from Soy bean) in 25 mL Erlenmeyer flask, phosphate buffer (100 mM pH 7.0) and enzyme. The reaction was carried out at 35 °C at 200 rpm. The samples were withdrawn at hour intervals of (24, 48, 72). The residual material was dried in vacuum and dissolved in 0.6 mL CDCl_3_/MeOH (2:1 *v*/*v*). The solvent also contains 20 mg of Triphenyl phosphate (TPP) as Internal Standard (IS) referencing (δ -17.8 ppm). The experiment was performed on a Bruker Avance III spectrometer (Karlsruhe, Germany). The data were processed by *MestReNova* software. The measurement took 7 min with automatic temperature adjustment. The analysis consists of phase correlation, baseline correction, and integration [19,20,21,22,23,24].

### 2.5. Protein Sequence and Structure Analysis

The TmPLB1 sequence was taken from Uniprot B6QH65_TALMQ. The sequencing were performed with PSI-BLAST [25] and HMMER [26]. Homology modelling was done to predict three dimensional structure of TmPLB1 using (PDB ID: 1FJ2) as the template [27,28,29]. The structure alignment were attained with fold recognition and applied to develop 3D model by Modeler 9.10 [27,30]. The reliable model was preferred on the basis of TM-score and DOPE profile. The selected model was improved with SCWRL 4.0 [31] and energy-minimized with GROMOS 43B1 force field executed in Deep View [32]. Lastly, the energy-minimized 3D model was assessed using VERIFY_3D [33] and ProSA [34]. The important catalytic residues and the phosphosites were predicted by KinasePhos server.

### 2.6. Molecular Dynamics Simulation

Molecular Dynamics Simulation were done on TmPLB1 at 30 °C, 35 °C, and 40 °C to examine its dynamics at the atomic level. First, 3D model of TmPLB1 were solvated in a cubic box of H_2_O molecules with a least distance of 1.0 nm among the TmPLB1 and the ends of box. The Simple Point Charge (SPC) model [35] were used. The system was neutralized with the addition of Na^+^ and Cl^−^ ions. In total, 67 Na^+^ and 59 Cl^−^ ions to a concentration of 0.15M of aqueous solution. The system was minimized through 1500 steps of steepest descent to increase the system temperature from 0 K to 303.15 K (30 °C), 308.15 K (35 °C), and 313.15 K (40 °C) through the equilibration period (100 ps) at a persistent capacity below periodic boundary setting. Equilibration were done in two steps: NVT ensemble (constant number of particles, volume, and temperature for 100 ps) and NPT ensemble (constant number of particles, pressure, and temperature for 100 ps).

In order to ensure molecular basis of TmPLB1 thermostability, molecular dynamics simulations were carried after equilibration phase for 100 ns at 30 °C, 35 °C, and 40 °C, respectively. GROMACS 5.1.2 with the all-atom OPLS/L force field were used to perform energy minimizations and MD simulations [36]. Using particle-mesh Ewald method to carry out electrostatic interactions with a space of 10 Å and van der Waals interactions truncated at 10 Å [37]. Using the velocity-rescaling thermostat to adjust temperature with 0.1 ps and pressure with Parrinello–Rahman barostat having time 2 ps [38,39].The neighbor list was upgraded at every 10 steps with a time integration of 2 fs. The bonds were controlled by LINCS [40] algorithm for the protein and the SETTLE [41] algorithm for H_2_O molecules. The subsequent courses were analyzed by GROMACS and 3D model were prepared using PyMOL [42].

## 3. Results and Discussion

The putative TmPlb1 gene sequence was collected from the genomic data of *Talaromyces marneffei strain* GD-0079 which is a dimorphic fungi. The gene consists of 732 bp ORF and encoded by 243 amino acids. The putative TmPLB1 shared higher similarity with other phospholipases enzyme carrying species such as *Talaromyces stipitatus* (91%), *Rasamsonia emersonii* (73%), *Aspergillus flavus* (67%), *Aspergillus fumigatus* (66%), *Saccharomyces cerevisiae* (40%), and with rats and humans (39%) (human acyl protein thioesterase 1 that have phospholipase and deacylation activity). In addition, secondary structure of TmPLB1 showed homogeneously distributed in helix and strand. However, due to lack of 3D structure of TmPLB1, we further performed structural analysis. Human acyl protein thioesterase 1(PDB: 1FJ2) was selected as suitable template with a 39.2% sequence identity. The template protein has a phospholipase and deacylation activity [42].

### 3.1. Recombinant TmPLB1 Expression and p Simple Point Charge (SPCPurification)

To evaluate whether this putative TmPLB1 has phospholipase activity, gene was artificially synthesized and expressed as His-tag fusion protein in *E. coli* Shuffle T7 expression system. He et al. 2016 performed expression and characterization of *Talaromyces marneffei* active phospholipase B using *Pichia pastoris* GS115 expression system. Using Ni-affinity chromatography, the protein concentration of active TmPLB1 was 240.4 mg L^−1^ of the culture medium [43]. It usually takes 5–7 days for expression of recombinant protein in *Pichia pastoris* expression system. The recombinant TmPLB1 showed maximum expression and was present in soluble fraction of cell lysates (Figure S1). Purified recombinant TmPLB1 had a predicted molecular weight of 35 KDa. The purification summary was mentioned in Table S1. Purified protein by Ni^+^ affinity chromatography produced 4.96 mg of protein with a total enzyme activity of 240 U that was enough for biochemical characterization and analysis of phospholipids in Lecithin (soybean) using enzymatic hydrolysis by ^31^P NMR for application evaluation.

### 3.2. Biochemical Characterization

#### 3.2.1. Optimum Temperature

**Figure 1:**
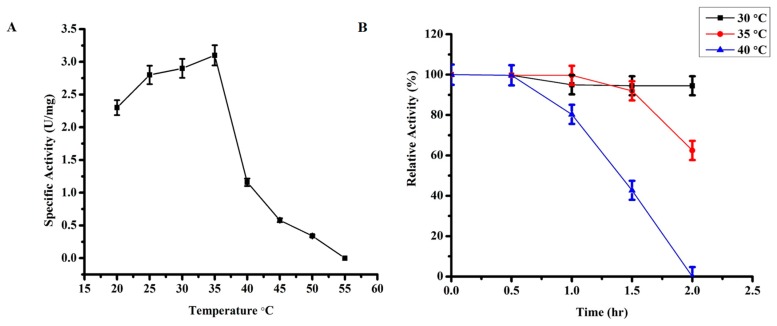
Effect of Temperature and thermostability on phospholipase B activity. (**A**) Optimum temperature of putative phospholipase B was assessed at temperature from 20–55 °C. (**B**) The thermostability of putative phospholipase B was carried out at 30–40 °C. The enzyme was more stable at 30 °C. The activity were assessed after every 30 min and the activity at zero is taken as 100 and is the maximum activity. The error bars shows the mean ±SD of the experiments in triplicate (*n* = 3).

Purified TmPLB1 was assessed at different temperatures ranges from 20–55 °C. The phospholipase activity were measured using emulsified Lecithin (Soybean). As shown in Figure 1A, TmPLB1 showed optimum temperature at 35 °C. The thermostability of TmPLB1 (Figure 1B) were investigated by incubation at (30 °C, 35 °C and 40 °C) for 2 h. Time interval was 30 min to measure the residual activity and initial activity was taken as 100%. The enzyme was stable at 30 °C. The enzyme had 50% activity when incubated at 35 °C for 2 h. The optimum temperature of the putative phospholipase B indicating that it is a thermolabile enzyme.

#### 3.2.2. Optimum pH

**Figure 2:**
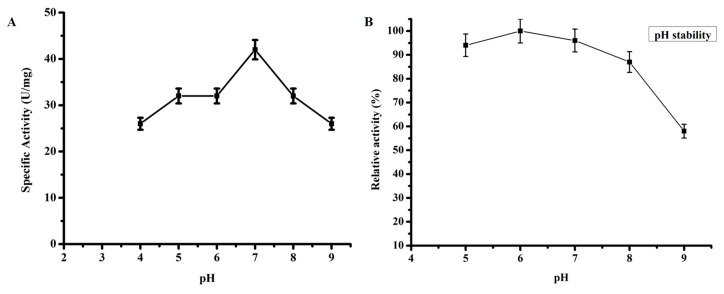
Effect of pH and its stability on phospholipase activity of TmPLB1. (**A**) The optimum pH of the enzyme were investigated from pH 4–9 using the buffers 100 mM citric acid-sodium citrate (pH 4.0 and 5.0), 100 mM phosphate buffer (pH 6.0, 7.0), 100 mM Tris-HCl (pH 8.0), and 100 mM Gly-NaOH (pH 9.0). The enzyme showed activity at pH 7.0. (**B**) The pH stability was assessed by incubating the enzyme at pH 5–9 in the stated buffers above for 12 h at 4 °C. The enzyme showed maximum pH stability at pH 6.0. The error bars indicates the mean ± SD of the experiment in triplicate (*n* = 3).

TmPLB1 showed optimum pH at 7.0 (Figure 2A) and has high activity at this neutral pH. It retained about 25–30% specific activity at pH 8.0 and 9.0. In order to investigate pH stability, (Figure 2B) the enzyme was incubated for 12 h with a pH ranges 5.0–9.0 at 4 °C. TmPLB1 had more than 90% of the enzyme activity at pH 6.0. The enzyme activity declined quickly at pH 9.0 with only 60% of relative activity, respectively.

#### 3.2.3. Effect of Chemicals on the Activity and Stability of TmPLB1

**Figure 3:**
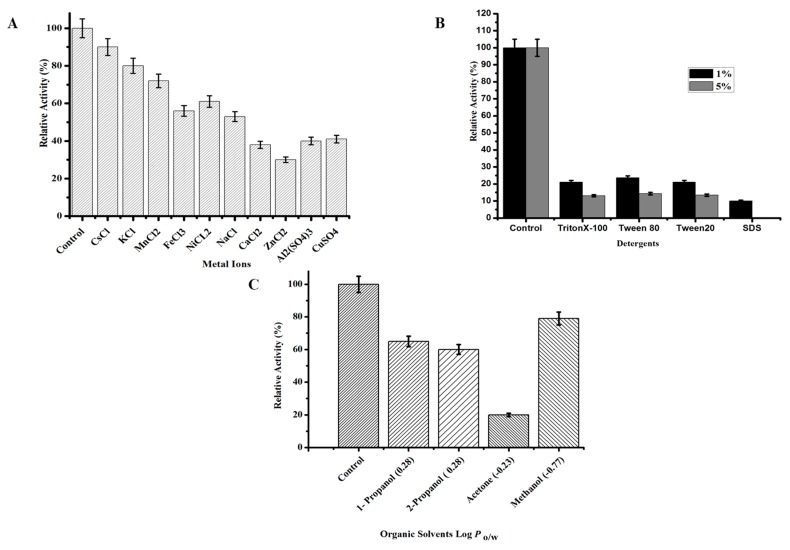
Effect of various chemicals on the activity and stability of phospholipase B TmPLB1. (**A**) Effect of metal ions (1 mM) on enzyme activity. (**B**) Effect of detergents on enzyme activity (1% and 5%). The TmPLB1 activity were determined using Lecithin (Soybean PC Emulsion). (**C**) Effect of organic solvents (10%) on enzyme activity. Log *P* values are the coefficient of solvent among *n*-octanol and water. It is used quantitatively to measure the polarity of organic solvent. The Log *P_o/w_* values are shown in braces. The data presented shows the mean ± SD (*n* = 3) of the experiments performed triplicate with respect to non-treated control samples. The samples without addition of any chemical was used as control and its activity was taken as 100%.

Metal ions (Figure 3A) (CsCl, KCl, MnCl_2_, FeCl_3_, NiCl_2_, NaCl, CaCl_2_, ZnCl_2_, Al_2_ (SO_4_)_3_, CuSO_4_) effects TmPLB1 activity and stability with 1mM concentration. The TmPLB1 showed high stability in Cs^+^, K^+^, ions with 80% activity while Mn^+2^, Fe^+3^ Na^+^ show weak inhibitory effect as shown in Figure 3A. Ca^+2^ and Zn^+2^ exhibited strong inhibitory effects, with the relative TmPLB1 activity falling below 40%. Metal ions are also proved to change the structure conformation of enzyme. This property is not reliable for substrate binding of enzyme. Doi and Nojima reported that lysophospholipase (Phospholipase B) activity was inhibited by 10 mM Fe^+3^, and Al^+3^. He also reported that Ca^+2^ also affects the phospholipase B activity and stability [4,44]. Divalent metal ions affects phospholipids by forming complexes and affects the hydration of phosphate group [4,45,46,47] (*p* > 0.05).

The activity of TmPLB1 was analyzed in the presence of 10% (*v*/*v*) concentration of organic solvents. As shown in Figure 3C, TmPLB1 maintained activity above 60% when treated with methanol, 1-propanol, and 2-propanol. In contrast, TmPLB1 was most susceptible to acetone with a drastic decrease in activity to around 20% (*p* > 0.05). Log *P_o/w_* values are determined to check the hydrophobicity and hydrophilicity of the solvents for TmPLB1. It is reported that higher Log *P_o/w_* may causes prevention of binding of enzyme and substrate. Hydrophilic solvents with low log *P_o/w_* values deactivates enzymes at unchanging level [48]. A unique phospholipase B from *Thermotoga lettingae* (TLPLB) showed significant organic solvent tolerance. This phospholipase B had more stability in non-polar solvent than in polar solvents. It attained 91%–161% its phospholipase B activity when incubated in hydrophobic organic solvents, instead its activity declined sharply in hydrophilic solvents from 10%–38% [1].

In this study we used anionic and non-ionic detergents. SDS an anionic detergent seriously affected the activity of TmPLB1. In contrast, non-ionic detergents (Tween 80 and Tween 20) do not change the activity of enzyme [49] as shown in Figure 3B. In the present study TmPLB1 maintained 20% and 10% relative activity with 1% and 5% (*w*/*v*) detergent concentration. The enzyme was completely inhibited with 5% sodium dodecyl sulphate (SDS) (*p* > 0.05). Detergents modify phospholipids at oil water interfaces and stops the access of substrate at catalytic site of enzyme. Activity of the enzyme by detergents can be inferred by the hydrophilic/lipophilic balance (HLB) at which a detergent is distributed amongst polar and non-polar phases. Lower amount of detergent causes the release of enzyme from the cells while higher deactivates it [48].

#### 3.2.4. Circular Dichroism Spectral Analysis

Circular Dichroism were performed for structural conformation and functional responses of a protein. Tyukhtenko et al., 2018 showed that CD spectra is indicative of the secondary structure prediction and no conformational change or folding may occurs which might be possible due to nonconservative substitutions at certain at certain length of spectra. Anwer *et al.,* 2016 reported that secondary structural changes of a protein can be assessed from CD spectra and the protein follow two state folding mechanism. A lack of fixed tertiary interactions could results in a conformational shift towards ensemble of fluctuating structures [16,17,18]. The CD was performed in the far-UV region (190–260 nm) to predict the secondary structure and stability of TmPLB1. The CD spectra is a characteristic of protein structure containing β-sheet and α-helices, at 209 and 222 nm (Figure S2 and Table 1). The spectra clearly showed that TmPLB1 have distinct secondary structure. From the results of Circular Dichroism and Molecular dynamic simulations, α- helix and β -turn were quite similar.

### 3.3. Identification of Standard Phospholipids by ^31^P NMR

Advanced analytical techniques are employed nowadays for the identification and quantification of compounds in complex mixtures. They cover several applications from metabolomics to identification of impurities. A number of these experiments showed the use of ^31^P NMR for identification and quantification of phospholipids in complex mixtures. ^31^P NMR separates phosphate buffers and phospholipids based on the chemical shifts from each other. So to differentiate peaks with chemical shifts (ppm) it is relatively important that resolution must be higher and the peaks narrow [20]. Lecithin (Soybean) is a natural emulsifier and a key component in food, cosmetic products and drug industry. It mainly consists of different phospholipids (PL) like phosphatidylcholine, phosphatidylethanolamine, phosphatidylinositol, phosphatidylserine, phosphotidylglycerol, and phosphatidic acid along with lyso-phospholipids like PC, PE, PI, PS, PG, and PA with other monoacylglycerols and vitamins too [21,22].

**Figure 4:**
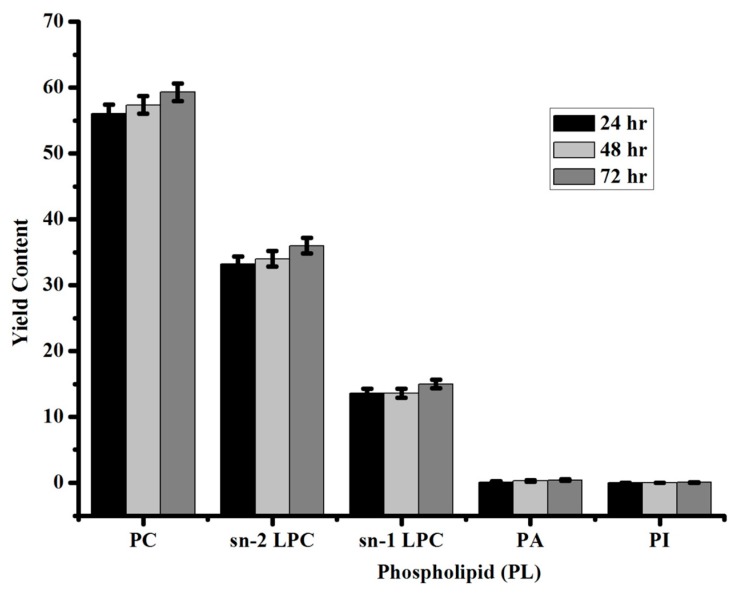
Analysis of the enzymatic hydrolysis of phospholipase B using Lecithin (Soybean) by ^31^P NMR. The experiment was done in triplicate at three different time intervals. The error bar represents the mean SD± of the experiment (*n* = 3).

In this study, Lecithin (Soybean) were used to confirm the hydrolytic activity of TmPLB1 at three different time intervals by ^31^P NMR (Figure 4). ^31^P NMR spectra in these results showed phosphatidylcholine, lysophosphatidylcholine, phosphatidic acid, and phosphatidylinositol with other minor phospholipids (Figure S3). During the enzymatic hydrolysis, the acyl residue migration occurs from the *sn-1* to *sn-2*. The different classes of phospholipids were quantified on the basis of peak integration as shown in Table 2. Our results are consistent with the previous results obtained by vegetable lecithin using ^31^P NMR spectroscopy [21]. Yang et al. demonstrated that the lyso-phospholipids obtained from phospholipase enzyme can have higher emulsification action. They did continuous enzymatic hydrolysis reaction using phospholipase at multiple time intervals and investigated phospholipid composition in commercial lecithin to obtain multiple classes of phospholipids by ^31^P NMR analysis. They also explained the actions of phospholipases on phospholipids during hydrolysis reaction, as how acyl residue migration takes place at different cleavage sites i.e., *sn-2* and *sn-1* [19]. Our present work is consistent with their work for particular characterization of phospholipids using Lecithin (Soybean) by enzymatic hydrolysis at different time intervals. By using this procedure, routine analysis for identification and classification of phospholipid (lyso-PLs) can be done without using standards for every class of phospholipids.

Several researchers found that hydrolysis of lyso- forms of phospholipids have many practical applications. But the complex mixture of Lecithins hydrolysis made it difficult for qualitative and quantitative analysis of phospholipids. A consistent procedure is required for the characterization of phospholipids in complex mixtures. ^31^P NMR proved to be an advanced analytical technique for the analysis of different classes of phospholipids in different industries [21,22].

### 3.4. Protein Sequence, Structure Analysis and Identification of Catalytic Triad

The template was identified by HHpred and PSI-Blast for homology modelling of TmPLB1. Target and template protein sequence contained several conserved functional residues. Based on TM-score and DOPE sketch, ten models were assessed and categorized. The side chain of model were refined by SCWRL4 package and further energy minimization were done to stop non-useful interactions using Deep View and GROMACS 5.1.2 [35]. The energy minimized model was more improved by means of Verify_3D, where a positive averaged 3D-1D score value > 0.2 specifies right folding and was a more reliable model with 80.66% of the residues. PDB sum was done to create topology of TmPLB1 structure for understanding structural features of the enzyme [50]. The predicted model contained 2 β-sheets that includes 7 mixed and 2 antiparallel β-strands, 7 α-helices, 3 β–α–β motifs, 1 β-bulge, 2 helix–helix interactions, 28 β-turns, and 4 gamma turn. The Ramachandran plot of the TmPLB1 suggested that 167 amino acid residues (85.2%) are in most favored region, 22 amino acid residues (11.2%) are in additional allowed regions, 6 amino acid residues (3.1%) are in a generously allowed region, and 1 amino acid residue (0.5%) is in a disallowed region.

**Figure 5:**
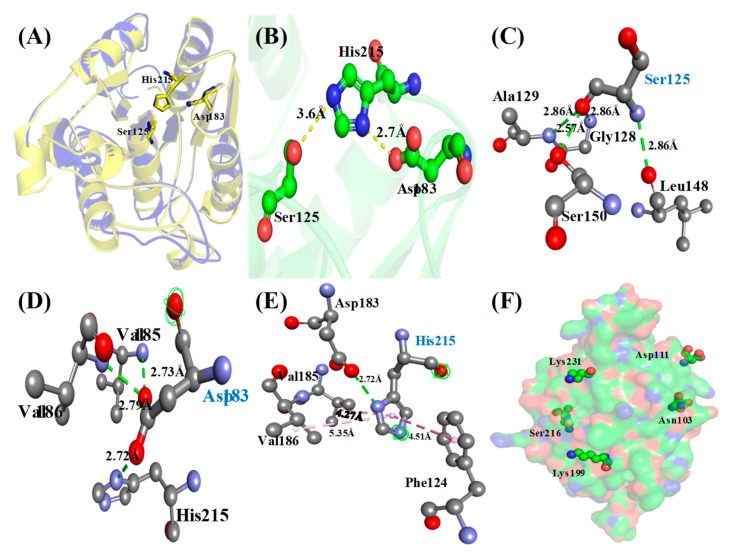
Homology model of TmPLB1 and identification of catalytic triads. (**A**) Homology model of TmPLB1 aligned with a template (PDB: 1FJ2). The proposed catalytic triad residues such as Ser125, Asp183, and His215 were shown in stick representation. (**B**) The arrangement of catalytic triads. The interactions of (**C**) Ser125, (**D**) Asp183, and (**E**) His215 with other amino acids near the active sites. Putative hydrogen bonds were indicated with green lines, while hydrophobic interactions were indicated with purple lines with distances labeled. (**F**) Putative phosphorylated sites on the surface of TmPLB1.

3D structure of the TmPLB1 confirmed the presence of a catalytic triad at a catalytic site with serine acting as a nucleophile, aspartic acid, and a conserved histidine residue. Structure alignments of TmPLB1 with its template indicated that its active site pockets were similar, with Ser125, Asp183 and His215 as the catalytic triad (Figure 5A). OG of Ser125 and NE2 of His215, as well as OD2 of Asp183 and ND1 of His215, were in proper distances to favor proton transfer (Figure 5B). Hydrogen bonds along the residues near active site were generated by Ser125, Asp183 and His215 which stabilized catalytic triad of TmPLB1 (Figure 5C–E). In addition, the imidazole side chain of His215 might be also involved in hydrophobic interaction with some residues (Figure 5E). Structure analysis showed that Asp183 with Ser125 and His215 established the catalytic triad of TmPLB1.

#### Identification of Phosphosites

Phosphosites are the sites present on protein associated with phosphorylation. Phospholipase B generates free fatty acids and lysophospholipids or glycerol-3-phosphodiesters by cleaving *sn-1* and *sn-2* position. Several studies described the importance of phosphosites in different classes of phospholipases. Protein phosphorylation is a quick changeable switch of protein function. It adds negative charge to amino acid side chains. The negative charged amino acids (Asp/Glu) may lessen the phosphorylated form of protein [51]. Phosphorylation of serine is crucial for initiation of cytosolic phospholipase A2 in vivo because overexpression in some cells fails to increase agonist-induced arachidonic acid release [52]. Protein phosphorylation is crucial in prokaryotes, in which kinases catalyze the phosphorylation of histidine residues [53]. Most eukaryotic proteins have more than one Phosphosites contains serine (89%), threonine (10%), and tyrosine (<1%) residues [51]. Phosphorylated proteins in a cell are essential for predicting link among enzymes and substrates. The template Human Acyl protein thioesterase have several reported phosphosites such as Lys92, Lys100, Lys185, Ser204, Ser205, and Lys219. In the predicted model of TmPLB1, residues Asn103, Asp111, Lys199, Ser216, Ala217, and Lys231 are present adjacent to phosphosites of template (Figure 5F) [51,52,53,54]. Phosphosite at Lys92 in template are replaced with Asn103 in the TmPLB1. The rest of the phosphosites are have similar amino acids in both the template and TmPLB1. Most of these Phosphosites are present on the surface of TmPLB1. Additionally, KinasePhos server predicted several other phosphorylation sites on TmPLB1 such as Thr18, Ser43, Tyr101, Ser125, Tyr181, and Tyr228 using sequence based Profile Hidden Markov Model [55].

### 3.5. Structural Deviations, Hydrogen Bonding, and SASA Analysis

**Figure 6:**
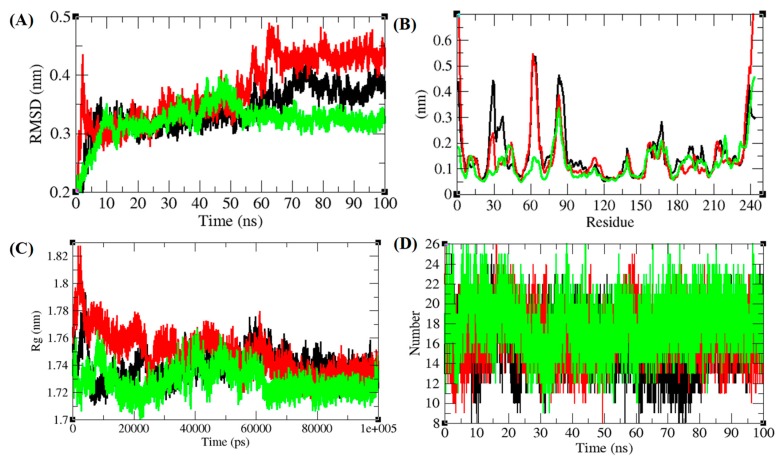
The structure nonconformities and residual variations of TmPLB1. (**A**) RMSD (Root mean square deviation) of TmPLB1 respect with time. (**B**) The backbone residues fluctuations (RMSF) of TmPLB1. (**C**) Radius of gyration (*Rg*) calculated the compactness of TmPLB1. (**D**) H-bonds shown amongst main chain plus side chain of TmPLB1. The black, red and green colors were representing the respective temperatures at 30 °C, 35 °C, and 40 °C, respectively.

The temperature influence has a strong effect on the conformational change of enzyme [56,57]. A fundamental property to check the protein stability matching experimental structure is confirmed by root mean square deviation (RMSD) [58]. The RMSD plot of TmPLB1 showed to have random fluctuations at 30 °C and 35 °C. These fluctuations might arise due to flexibility of TmPLB1 structure and it is responsible for its biological functions (Figure 6A). The vibrations and equilibrium of the protein are not random and depends on the flexibility of structure. The root mean square fluctuation (RMSF) of the TmPLB1 at 30 °C, 35 °C, and 40 °C were plotted to check residue number in order to calculate average fluctuation of residues during simulation (Figure 6B). Further radius of gyration (*Rg*) assessment was done to understand stability of protein in biological system. Least tight packing of protein is supposed to have higher radius of gyration. R*g* plot supposed that the TmPLB1 has tight packing at 40 °C. It may be responsible for loss of function at this temperature (Figure 6C). The overall results suggested that the flexibility of TmPLB1 structure is more at 30 °C and 35 °C.

**Figure 7:**
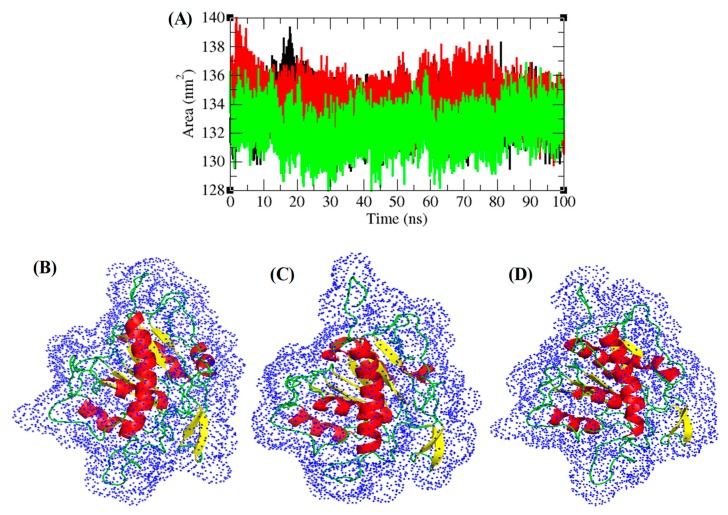
SASA (Solvent Accessible Surface Area) of TmPLB1. (**A**) SASA of TmPLB1 were represent in cartoon model (**B**) 30 °C, (**C**) 35 °C, and (**D**) 40 °C, respectively. Black, red, and green colors were depicted the 30 °C, 35 °C, and 40 °C, respectively.

Hydrogen bonding between main chain and side chain provides important information regarding protein stability. The hydrogen bonds among key chain and side chain of TmPLB1 were solved in solvent environment through the 100 ns Molecular dynamics simulations. Average hydrogen bonds were 16 ± 2.5, 17 ± 2.3, and 15 ± 2.5 at 30 °C, 35 °C, and 40 °C, respectively (Figure 7D). The hydrogen bond plots suggested that these temperatures have no effects on unfolding and folding of TmPLB1. The solvent accessible area (SASA) interconnects solvent molecules in surface area of protein [59]. The average SASA values for TmPLB1 were found to be 134 ± 1.3 nm^2^, 134 ± 1.2 nm^2^, and 132 ± 1.2 nm^2^ at 30 °C, 35 °C, and 40 °C, respectively. The normal Solvent Accessible Surface Area (SASA) for TmPLB1 were found to be lower at 40 °C. It can be assumed that internal or active residues in the TmPLB1 are not visible to solvent at 40 °C (Figure 7).

### 3.6. Secondary Structure Analysis

**Figure 8:**
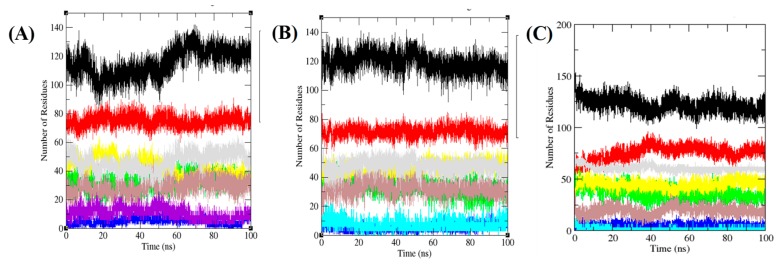
Secondary structure plot. Graphical representation indicating the structural elements present in the TmPLB1 during 100 ns MD simulations at (**A**) 30 °C, (**B**) 35 °C, and (**C**) 40 °C, respectively. The structure represents α-helices, β-sheets, β-bridge and turns. The black, red, green, blue, yellow, brown, grey, and violet color represent overall structure, coil, β-sheet, β-bridge, bend, turn, α-helix, and 3-helix, respectively.

The structural contents of a protein can be seen at multiple temperature to ensure changes at different temperature. The protein secondary structure contains α-helix, β-sheet broken into single residues at every phase. The residues involved in predicted structure makeup of TmPLB1 at altered temperature were calculated (Figure 8, Table 3). The average percentage residues joined through the 100 ns MD simulations were 48%, 49%, and 44% at 30 °C, 35 °C, and 40 °C, respectively. Less percentage residues were participated at 40 °C suggested that the unfolding of TmPLB1 might takes place at this temperature. While the TmPLB1 showed stable conformations at 30 °C and 35 °C. As the temperature increases from 35 °C to 40 °C, the denaturation of TmPLB1 takes place due to decrease in α-helix and increase in bends. The stability of TmPLB1 at 30 °C and 35 °C are due to higher number of residues joined in formation of α-helix and β-sheets.

### 3.7. PCA and Gibbs Free Energy Landscape

**Figure 9:**
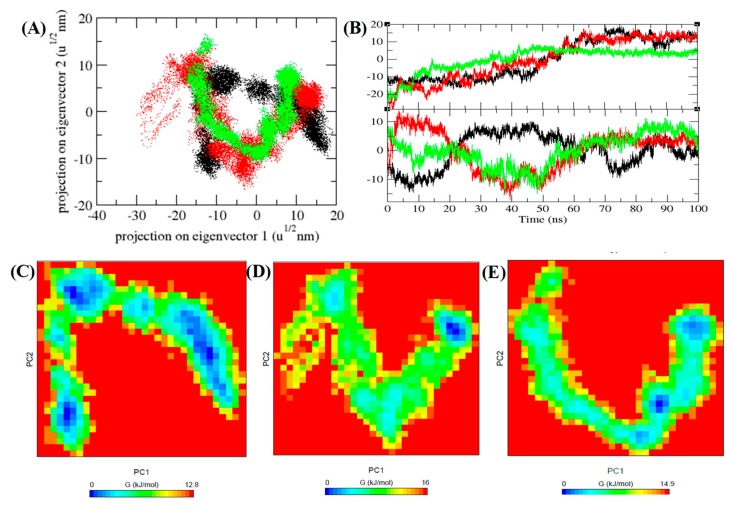
PCA and Gibbs free energy analysis. (**A**) Different estimates at different temperatures of eigenvectors. (**B**) The trajectories on eigenvectors with respect to time. The Gibbs energy plot obtained for TmPLB1 at different temperatures (**C**) 30 °C, (**D**) 35 °C, and (**E**) 40 °C, respectively.

Motion of the protein during the course of simulation is shown by principal component analysis (PCA) [60]. Considering the backbone as reference the dynamics of TmPLB1 was calculated using *gmx covar* function integrated in GROMACS. Average proteins motion were obtained as PCs (PC1, PC2, and PC3) [61]. Addition of eigenvalues is a total motility in the system to link the flexibility of a protein below dissimilar environments. Eigenvalues and eigenvectors for TmPLB1 at 30 °C, 35 °C, and 40 °C were 323.02 nm^2^, 326.21 nm^2^, and 171.25 nm^2^ were calculated. Eigenvalues for TmPLB1 at 40 °C was found to be very low as compared to other temperatures. This might be the possibility of low activity of TmPLB1 at 40 °C. Further, the projections of trajectories on eigenvectors displayed differences in the position of atoms at different temperatures during the MD simulations (Figure 9A,B).

First two PCs were then used as input to calculate the Gibbs free energy landscape. The color free energy landscape inspect direction of fluctuation for all Cα atoms of the TmPLB1 from 100 ns trajectory at 30 °C, 35 °C, and 40 °C, respectively. The parallel free energy contour map having deep blue color representing lower energy. It has been showed that the key energy in universal free energy lowest region were different. These free energies well indicate stable conformational states of molecule. The TmPLB1 has deeper blue energy global minima at 30 °C (Figure 9C). It indicates a stable conformation of TmPLB1 during 100 ns MD simulations.

## 4. Conclusions

In this study, a putative phospholipase B from *Talaromyces marneffei* GD-0079 (TmPLB1) was biochemically characterized. The detailed structure analysis by computational approaches have been performed in order to study its conformational profile. TmPLB1 is a thermolabile enzyme with optimum activity at 35 °C and pH 7.0. The hydrolysis of TmPLB1 using Lecithin by ^31^P NMR showed PC and lyso-PL as the major phospholipids. This work showed that the low temperature phospholipase B can be employed in food industry to determine phosphorus components in food. The phospholipids can also be modified from one form to another. This key factor can be employed in food industry for characterization of phospholipids.

## Figures and Tables

**Table 1:** Estimated data of a predicted secondary structure of TmPLB1 by CDNN software.

	185–260 nm	190–260 nm	195–260 nm	200–260 nm	205–260 nm	210–260 nm
**Helix**	20	20	12	3	2	1
**Antiparallel**	2	1	8	15	16	22
**Parallel**	14	15	17	22	25	20
**β-turn**	10	10	13	13	15	18
**Random coil**	54	54	50	47	42	39
**Total Sum**	100%	100%	100%	100%	100%	100%

**Table 2:** Analysis of the enzymatic hydrolysis of phospholipids in Lecithin (Soy bean) by ^31^P NMR.

Phospholipid (PL)	Yield Content
Phosphotidylcholine (PC)	59 ± 1
*sn-2* lysophosphatidylcholine (2-LPC)	36 ± 1
*sn-1* lysophosphatidylcholine (1-LPC)	15 ± 0.6
Phosphatidic acid (PA)	0.4 ± 0.1
Phosphatidylinositol (PI)	0.05 ± 0.01

**Table 3:** Percentage of residues participated in average structure formation.

Secondary Structure (SS%)								
Temp	Structure *	coil	β-Sheet	β-Bridge	Bend	Turn	α-Helix	3-Helix
30 °C	48%	31%	14%	2%	17%	12%	19%	4%
35 °C	49%	29%	14%	2%	19%	14%	20%	3%
40 °C	44%	30%	13%	4%	21%	10%	17%	5%

* Structure = α-helix + β-sheet + β-bridge + Turn.

## Supplementary Materials

The following are available online at https://www.mdpi.com/2218-273X/10/2/231/s1. Figure S1. Purification of phospholipase B TmPLB1 by nickel-chelate chromatography and eluted with washing buffer 50 mM phosphate buffer (PB), (pH 7.4) that contained 500 mM imidazole. **KDa** (molecular weight of the protein marker in kilo daltons). **Lane M** (molecular weight of marker). **Lane 1**. Purified TmPlb1. Figure S2. Far-UV CD spectra of TmPLB1. The protein was incubated at 25 °C in 20 mM sodium phosphate buffer (pH 7.4) at a concentration of 0.5 mg/mL Unfolding was followed by monitoring the CD signal at 222 nm. Figure S3. Rapid Identification and classification of phospholipids in ^31^P NMR by enzymatic reaction mechanism of TmPLB1 at three different time intervals (A) 24 hr, (B) 48 hr, and (C) 72 hr with internal standard Triphenyl Phosphate (TPP). Table S1. Overview of TmPLB1 purification.

## Abbreviations

(FFAs) Free Fatty acids, (NMR) Nuclear magnetic resonance, (TmPLB1) *Talaromyces marneffei* phospholipase B1, (lyso-PLs) Lysophospholipids, IPTG (*Isopropyl β-D-1-thiogalactopyranoside*), (TPP) Triphenyl phosphate, (MD simulation) Molecular dynamics simulation, PC (phosphatidylcholine), (RMSD) Root mean square deviation, (RMSF) Root mean square fluctuation, (SASA) Solvent accessible area, (PCA) Principal component analysis.
